# The needs of people with dementia living at home from user, caregiver and professional perspectives: a cross-sectional survey

**DOI:** 10.1186/1472-6963-13-43

**Published:** 2013-02-04

**Authors:** Claudia Miranda-Castillo, Bob Woods, Martin Orrell

**Affiliations:** 1 Escuela de Psicología, Facultad de Medicina, Universidad de Valparaíso, Av. Brasil 2140, Valparaíso, Chile; 2 Dementia Services Development Centre, Bangor University, 45 College Road, Bangor, LL57 2AS, UK; 3 Unit of Mental Health Sciences, University College London, London, 67-73 Riding House Street, London, W1W 7EJ, UK

**Keywords:** Needs assessment, Dementia, Caregiver, Community-dwelling

## Abstract

**Background:**

Few reports have been published about differences in perspectives on perceived needs among community-residing people with dementia, their family caregivers, and professionals. The aim of this study was to compare these perspectives.

**Method:**

During 2006 and 2007, one-hundred and fifty two interviews of people with dementia and their caregivers about the needs of the person with dementia were performed by four professionals using The Camberwell Assessment of Need for the Elderly (CANE). Professionals’ views on met and unmet needs of people with dementia were obtained for the total sample, family caregivers’ perspectives were gained for 125 people with dementia, and people with dementia’s views on their own needs were obtained for 125 persons with dementia.

**Results:**

People with dementia reported fewer needs compared with the reports of their caregivers and the professionals. The most frequent unmet needs reported by people with dementia, caregivers and professionals were in the areas of daytime activities, company, and psychological distress; however, people with dementia rated psychological distress as the commonest unmet need.

**Conclusions:**

Since the priorities of people with dementia can be different from those of caregivers and professionals, it is important to consider all perspectives when making care plans. Thus, compliance with treatment of people with dementia and also their quality of life could be potentially improved by a more collaborative partnership with them.

## Background

Since the concept of need is somewhat subjective, good practice requires that assessment of needs include not only the judgement of professionals but also the perspectives of users and caregivers [1]. In addition, needs assessment must be comprehensive, that is, it must include not only physical, but also social and psychological functioning [2,3].

In residential homes, Hancock *et al*. [4] assessed the needs of people with dementia considering resident, caregiver, staff and researcher ratings of need. They found that the most frequent unmet needs identified by researchers were in the areas of daytime activities (76%), psychological distress (48%) and company (41%). In the same study, Orrell *et al*. [5] also found that users (people with dementia) rated fewer needs as met than caregivers and staff. Staff reported more needs as met than family caregivers and family caregivers rated more needs as unmet than staff. Regarding the needs of people with dementia living in the community, studies show that most reported unmet needs are in the areas of daytime activities, company, memory, information and psychological distress. In The Netherlands, van der Roest *et al*. [6] interviewed 236 community-dwelling persons with dementia and 322 informal caregivers. Results showed that the most common unmet needs reported by people with dementia were memory (10.2%), information (9.9%) and psychological distress (4.9%). The most frequent unmet needs reported by caregivers were memory (32.5%), daytime activities (16.0%) and company (13.1%). Caregivers reported significantly more met and unmet needs than the person with dementia. In a recent study carried out in Chile by Covarrubias *et al*. [7], information (34.5%), company (28%) and daytime activities (25.2%) were reported by people with dementia as the most common unmet needs whereas, similar to van der Roest’s findings, daytime activities (40%), company (36.8%) and memory (36.6%) were the most frequent unmet needs reported by caregivers. It also has been found that younger people with dementia, those with a lower self-reported quality of life and higher number of behavioural and psychological symptoms, and those whose caregivers were not married reported higher levels of self-rated unmet needs [8].

In a recent review about the subjective experiences of persons with dementia, von Kutzleben *et al*. [9] found that, across different studies, the most common needs reported by people with dementia focused on the following issues: “emotions”, “losses and restrictions”, “to be taken seriously/being understood”, “emotional, social and practical support”, “maintaining normality”, “dementia as a challenge for relationships”, “coming to terms with the disease” and “disclosure of diagnosis”. Similarly, in another review, van der Roest *et al*. [10] found that the most frequent subjective needs reported by people with dementia were: “coping with the disabilities due to dementia”; “accepting the disease and the support and information offered”; and “the ability do the things they used to do”. Other needs related to wellbeing, such as the need “to be accepted the way they are” and the need for “social contact”, were reported as significant by people with dementia. Finally, another literature review [11] pointed out that people with dementia wanted to receive more information about the disease and to preserve their autonomy for as long as possible. Also, factors such as general well-being, participation in activities, having relationships with others and being useful were considered by people with dementia as important for their quality of life.

The aim of this study was to compare perceived needs according to users, their caregivers, and professionals in a sample of people with dementia living at home. This is the first study that includes professionals’ views about the needs of people with dementia living in the community. It was hypothesised that people with dementia would report fewer unmet needs than their caregivers or a professional.

## Method

The study was a cross-sectional survey carried out during 2006 and 2007. A total of 152 people with dementia living at home and 128 informal caregivers were recruited from health and social services, and voluntary organisations in North East London, Cambridgeshire and Liverpool. People with dementia were included in the study if they were aged 60 or over, had a diagnosis of dementia according to DSM-IV, and were living at home (not in institutions). A person was considered an informal caregiver when he/she was knowledgeable about the person with dementia and spent a minimum of 4 hours a week in direct contact with them.

### Recruitment of participants

The manager or appropriate member of the staff at the recruited centre was requested to make a first approach either with the caregivers of the people with dementia or the people with dementia themselves (depending on dementia severity and/or living situation) to give them the Information Sheet and to discuss if they were willing to be approached by the interviewer regarding this study. Participants who had no objection were contacted by the interviewer by phone and were given more information about the study as required. In addition, details of people who had attended the centre (e.g., person with dementia’s name, caregiver’s name, address, phone) were provided and a letter was sent to the caregiver and/or the person with dementia including Information Sheets about the study. One week later, the interviewer contacted them by phone, answered any questions they might have, explained the study, and looked for their willingness to participate. If the potential participant agreed to be involved, the interviewer arranged a day to carry out the interview at their homes.

### Interviews

Interviews were carried out by experienced professionals: a clinical psychologist (who was the main researcher) and three old age psychiatrists who were trained by MO, one of the authors of the Camberwell Assessment of Need for the Elderly (see description below) to undertake the assessment. Pilot interviews were discussed and agreement in rating criteria was achieved. Meetings with interviewers were scheduled once a month to discuss any difficulties that could have arisen during the interviews. All the interviews with people with dementia were carried out at their homes. Some interviews with caregivers were undertaken either at the home of the person with dementia, their own home, or at a health centre (e.g., memory clinic, day hospital).

### Consent procedure

The study was carried out in accordance with the latest version of the Helsinki Declaration. Ethical approval was granted by East London & the City HA Research Ethics Committee. All participants gave informed consent. Once the interviewer was at the home of the person with dementia or the caregiver, they answered any further queries about the Information Sheets and the study and sought informed consent as follows: written consent by signing a Consent Form was required from people with dementia and their caregivers. To achieve this, the interviewer approached them to explain the study again and to inform them about their right to withdraw at any time. Some people with dementia (11, 7.3%) were unable to provide written consent. When this occurred, the interviewer sought their assent (verbal consent). During this process, the interviewer made sure that he/she had taken as much time and care in explaining the information about this research as simply as possible. The interviewer avoided using long sentences and attempted to reduce any distractions. To find out if the participants had understood the information given, the interviewer observed their ability to ask any relevant questions and requested the participant repeat back the information and how it would relate to them. In addition, the interviewer clarified any doubts about the study and reiterated their right to withdraw at any time.

### Measure

#### Camberwell Assessment of Need for the Elderly (CANE)

The CANE [12,13] is a comprehensive tool which offers a structured evaluation of needs in older people. The instrument covers 24 areas of social, psychological, physical, and environmental needs related to the person with dementia. The instrument is based on the notion that identifying a need implies identifying a problem and a potential intervention to solve that problem and consequently meet the need. The CANE assesses whether there is currently a need in each specific area. Needs are rated as no need (score 0), met need (score 1), unmet need (score 2) or unknown (score 9) for each area. The CANE has shown good levels of reliability (α = 0.99) and validity (correlated with the CAPE-BRS, r = 0.66; and the Barthel, r = −0.53) [12]. Furthermore, this tool assesses the needs of older people from the perspectives of the person with dementia, the caregiver, the staff, and the professional, which allows comparison of these different views. The CANE is not a self-administered tool. The professional interviewed the person with dementia, listened to their views, and rated the user’s section of the CANE. The same procedure was carried out with the caregiver and finally the professionals made their own ratings as a result of the balance between their own perspectives and those of the caregiver and the person with dementia.

### Data analysis

Statistical analyses were undertaken using the SPSS 15.0 software package [14]. In general the significance level used was p < 0.05. Since the needs were not normally distributed among users, caregivers, and professionals, the Wilcoxon Signed-Rank Test was used to assess differences in the number of needs (met and unmet) reported by different informants. For a better understanding of the results, means instead of ranks are reported.

To assess agreement among professionals, caregivers, and users, Cohen’s Kappa coefficient (K) was used. Following recommendations given by Sim & Wright [15], the maximum attainable kappa (K_m_) was calculated. K_m_ is defined by Cohen [16] as *“the maximum value of kappa permitted by the total marginals”*. It *“reflects the extent to which the raters’ ability to agree is constrained by pre-existing factors that tend to produce unequal marginal totals, such as differences in their diagnostic propensities or dissimilar sensitivity in the tools they are using”*[15]. K/K_m_ (which indicates how much of the maximum possible agreement was actually achieved by the raters) was calculated to obtain the proportion of agreement achieved by the raters, considering the maximum kappa value [16,17]. The level of agreement was interpreted according to K/K_m_, where K/K_m_ ≥0.6 was considered as an indicator of a good level of agreement. All analyses performed to compare the different perspectives on needs were undertaken using the dyads for whom there was complete data on the particular comparison.

## Results

### Participants

Table 1 shows the demographic characteristics of the people with dementia. The mean age of people with dementia was 79.2 years (s.d. 6.8 years). There were 74 (48.7%) males and 78 (51.3%) females. The total sample of people with dementia had a mean MMSE score of 19.13 (s.d. 7.2), indicating mild/moderate cognitive impairment.

**Table 1:** Demographic characteristics of the subjects with dementia

**Demographic**	**Characteristic**	**%**
Age (years)	60-64	2.6
	65–79	42.8
	80-94	54.6
Gender	Male	48.7
	Female	51.3
Ethnicity	White	98.7
	Black	0.7
	Asian	0.7
First Language	English	97.3
	Other	2.7
Level of Education	Higher Education	20.3
	Secondary	75.6
	Below Secondary	4.1
Marital Status	Single	2.0
	Married/Living with a partner	55.3
	Separated/Divorced	4.6
	Widowed	38.2
Living Situation	Lives Alone	32.9
	Lives with Others	67.1
Has a Caregiver	Yes	90.1
	No	9.9

Most of the caregivers were older people (67.5%), women (71.1%), and married (89.8%). The majority (79, 66.9%) were caring for their relative 24 hours a day followed by 31 (26.2%) who spent from 4 to 20 hours a week looking after the person with dementia (see Table 2).

**Table 2:** Demographic characteristics of caregivers

**Demographic**	**Characteristic**	**%**
Age (years)	40-64	46.3
	65–89	52.8
	90-100	0.8
Gender	Male	28.9
	Female	71.1
Marital Status	Single	4.7
	Married/Living with a partner	89.8
	Separated/Divorced	3.9
	Widowed	1.6
Caregiver Relationship	Spouse	64.1
	Children	30.5
	Other relative	3.9
	Friend	0.7
Co-resident Caregiver	Yes	74.0
	No	26.0

Although we tried to interview all people with dementia with the CANE, 27 (17.8%) were unable to understand the questions, so for these participants only caregiver and professional descriptions in the CANE are available. People with dementia in this group were significantly more cognitively impaired (M = 5.4, s.d. 7.1) than the rest of the sample (M = 20.1, s.d. 6.2) (U = 116, p < 0.01). Also, those who had only caregiver and professional descriptions were significantly more functionally impaired (M = 3.1, s.d. 1.8) than the rest of the sample (M = 6.8, s.d. 3.8) (U = 196.5, p < 0.01). There were no differences in other person with dementia and caregiver factors (including demographics, social and clinical variables) between those who had only caregiver and professional descriptions in the CANE and the rest of the sample. In addition, ratings on the needs of people with dementia were obtained for only 125 caregivers. Fifteen (9.9%) persons with dementia did not have a family caregiver at all and 12 (7.9%) of them had a caregiver who did not meet the inclusion criteria.

### Needs rated by professionals

Professionals’ ratings on the CANE were available for all participants (n = 152). The mean total number of needs was 10.0 (s.d. 3.3, range 3–19), and of these 7.38 were met needs (s.d. 2.8, range 0–17) and 2.64 were unmet needs (s.d. 2.5, range 0–11). The most frequent met needs were memory (143, 94.1%), food (123, 80.9%) and money (117, 77%). The most common unmet needs were daytime activities (77, 50.7%), company (60, 39.5%) and psychological distress (47, 30.9%).

### Needs rated by caregivers

One hundred and twenty five caregivers completed the CANE. The mean total number of needs reported by caregivers was 10.1 (s.d 3.3, range 3–19), and of these 7.94 were met needs (s.d. 2.8, range 1–17) and 2.14 were unmet needs (s.d. 2.3, range 0–11). The most frequent met needs were memory (117, 93.6%), looking after the home (108, 87.1%), and food (106, 86.2%). The most common unmet needs were daytime activities (51, 41.1%), company (37, 29.8%) and psychological distress (33, 26.6%).

### Needs rated by people with dementia (users)

Users’ ratings on the CANE were obtained from 125 persons with dementia. The mean total number of needs reported by them was 6.58 (s.d. 3.5, range 0–17), and of these 5.41 were met needs (s.d. 2.6, range 0–12) and 1.17 were unmet needs (s.d. 1.8, range 0–10). The most common met needs were food (93, 75%), memory (90, 71.4%) and looking after the home (79, 63.2%). The most frequent unmet needs were psychological distress (27, 21.6%), daytime activities (18, 14.5%) and company (16, 12.8%). None of the people with dementia reported unmet needs in the areas of food, self-care, behaviour, alcohol, or benefits (financial assistance).

### Comparison of total number of needs by different informants

Overall, people with dementia reported significantly fewer met and unmet needs compared with caregivers (T = 112.5, p < 0.01; T = 122, p < 0.01, respectively) and professionals (T = 199.5 p < 0.01; T = 92, p < 0.01, respectively). Caregivers identified significantly more met needs than professionals (T = 295, p < 0.05). Professionals rated significantly more unmet needs compared with caregivers (T = 105, p < 0.01).

### Comparing professional and user ratings of individual areas of needs

The average K/K_m_ was 0.90, which indicates that 90% of the maximum possible agreement between professionals and users was reached (see Table 3). All the areas of the CANE reached a K/K_m_ value over 0.60, indicating a good agreement between professionals and users. Table 3 shows that users and professionals reached 100% of the maximum possible agreement in the areas of psychotic symptoms, deliberate self-harm, abuse/neglect, intimate relationship, and money. Professionals rated higher needs (met and unmet) in most of the areas of the CANE. They rated much higher numbers of met needs for memory, looking after the home, money, physical health, drugs, and self-care; and much higher unmet needs for daytime activities, company, psychological distress and eyesight and hearing. People with dementia only reported higher unmet needs for information.

**Table 3:** Professional and user ratings of need in individual CANE areas

**Professional (rater) vs user (n = 125)**	**Rater met need (%)**	**User met need (%)**	**Rater unmet need (%)**	**User unmet need (%)**	**K**	**K/ K**_**m**_
Accommodation	8 (6.4)	6 (4.8)	11 (8.8)	6 (4.8)	0.65**	0.87
Looking after home	96 (76.8)	79 (63.2)	10 (8.0)	3 (2.4)	0.53**	0.91
Food	102 (81.6)	93 (75.0)	7 (5.6)	0 (0.0)	#	#
Self-Care	68 (54.4)	42 (33.6)	11 (8.8)	0 (0.0)	#	#
Caring for another another	0 (0.0)	0 (0.0)	0 (0.0)	0 (0.0)	#	#
Daytime Activities Activities	36 (28.8)	22 (17.6)	68 (54.4)	18 (14.5)	0.21**	0.66
Memory	117(93.6)	90 (72.0)	7 (5.6)	3 (2.4)	#	#
Eyesight/Hearing Hearing	36 (28.8)	35 (28.0)	29 (23.2)	15 (12.0)	0.68**	0.85
Mobility	41 (32.8)	32 (25.6)	14 (11.2)	4 (3.2)	0.64**	0.91
Continence	23 (18.4)	15 (12.0)	8 (6.4)	3 (2.4)	0.66**	0.96
Physical Health Health	86 (68.8)	61 (48.8)	6 (4.8)	6 (4.8)	0.61**	0.97
Drugs	84 (67.2)	58 (46.8)	9 (7.2)	2 (1.6)	0.51**	0.98
Psychotic Symptoms	10 (8.0)	6 (4.8)	10 (8.0)	5 (4.0)	0.68**	1.00
Psychological Distress	23 (18.4)	20 (16.0)	44 (35.2)	27 (21.6)	0.61**	0.85
Information	27 (21.6)	11 (8.8)	12 (9.6)	16 (12.8)	0.50**	0.71
Deliberate Self-Harm Self-Harm	3 (2.4)	1 (0.8)	8 (6.4)	7 (5.6)	0.83**	1.00
Accidental Self-Harm Self-Harm	28 (22.4)	12 (9.6)	19 (15.2)	7 (5.6)	0.42**	0.86
Abuse/Neglect	11 (8.8)	3 (2.4)	4 (3.2)	1 (0.8)	0.40**	1.00
Behaviour	7 (5.6)	3 (2.4)	5 (4.0)	0 (0.0)	#	#
Alcohol	3 (2.4)	2 (1.6)	3 (2.4)	0 (0.0)	#	#
Company	15 (12.0)	10 (8.0)	52 (41.6)	16 (12.8)	0.37**	0.84
Intimate Relationships	4 (3.2)	3 (2.4)	11 (8.8)	6 (4.8)	0.77**	1.00
Money	96 (76.8)	67 (53.6)	2 (1.6)	1 (0.8)	0.51**	1.00
Benefits	11 (8.8)	5 (4.0)	1 (0.8)	0 (0.0)	#	#

**p < 0.01; * *p* < 0.05; K = Kappa; K_m_ = Maximum attainable kappa; K/K_m_ = Maximum agreement reached.

# Kappa coefficient could not be calculated because of insufficiently spread data.

### Comparing professional and caregiver ratings of individual areas of needs

The average K/K_m_ was 0.98, which indicates that 98% of the maximum possible agreement between professionals and caregivers was reached (see Table 4). All the areas of the CANE reached a K/K_m_ value over 0.80, indicating a good agreement between professionals and caregivers. Table 4 shows that caregivers and professionals reached 100% of the maximum possible agreement in most areas of the CANE, such as accommodation, looking after home, food, self-care, mobility, continence, drugs, psychotic symptoms, information, deliberate self-harm, abuse/neglect, behaviour, alcohol, intimate relationships, money and benefits. Professionals rated slightly fewer met needs and a higher number of unmet needs for most areas of the CANE. A slightly higher number of unmet needs were rated by professionals for food, physical health, information, and benefits. Caregivers identified slightly higher unmet needs only for information.

**Table 4:** Professional and caregiver ratings of need in individual CANE areas

**Professional (rater) vs caregiver (n = 125)**	**Rater met need (%)**	**Caregiver met need (%)**	**Rater unmet need (%)**	**Caregiver unmet need (%)**	**K**	**K/ K**_**m**_
Accommodation	8 (6.5)	9 (7.3)	11 (8.9)	10 (8.1)	0.97**	1.00
Looking after home	104 (83.9)	108 (87.1)	7 (5.6)	2 (1.6)	0.84**	1.00
Food	108 (87.1)	106 (86.2)	4 (3.2)	4 (3.2)	0.97**	1.00
Self-Care	70 (56.5)	72 (58.1)	12 (9.7)	9 (7.3)	0.96**	1.00
Caring for another another	0 (0.0)	0 (0.0)	0 (0.0)	0 (0.0)	#	#
Daytime Activities Activities	40 (32.3)	42 (33.9)	64 (51.6)	51 (41.1)	0.76**	0.89
Memory	116 (93.5)	117 (94.4)	7 (5.6)	7 (5.6)	0.85**	0.85
Eyesight/Hearing Hearing	34 (27.4)	37 (29.8)	27 (21.8)	25 (20.2)	0.93**	0.97
Mobility	43 (34.7)	47 (37.9)	10 (8.1)	6 (4.8)	0.94**	1.00
Continence	29 (23.4)	29 (23.4)	6 (4.8)	6 (4.8)	1.00**	1.00
Physical Health Health	81 (65.3)	80 (64.5)	7 (5.6)	6 (4.8)	0.95**	0.98
Drugs	84 (67.7)	86 (69.4)	9 (7.3)	6 (4.8)	0.95**	1.00
Psychotic Symptoms	12 (9.7)	13 (10.5)	11 (8.9)	9 (7.3)	0.95**	1.00
Psychological Distress	23 (18.5)	26 (21.0)	36 (29.0)	33 (26.6)	0.93**	0.97
Information	28 (22.6)	25 (20.2)	10 (8.1)	13 (10.5)	0.95**	1.00
Deliberate Self-Harm Self-Harm	3 (2.4)	3 (2.4)	6 (4.8)	6 (4.8)	1.00**	1.00
Accidental Self-Harm Self-Harm	30 (24.2)	30 (24.2)	15 (12.1)	13 (10.5)	0.94**	0.97
Abuse/Neglect	10 (8.1)	10 (8.1)	2 (1.6)	2 (1.6)	1.00**	1.00
Behaviour	9 (7.3)	9 (7.3)	5 (4.0)	5 (4.0)	1.00**	1.00
Alcohol	3 (2.4)	3 (2.4)	3 (2.4)	3 (2.4)	1.00**	1.00
Company	14 (11.3)	17 (13.7)	44 (35.5)	37 (29.8)	0.87**	0.97
Intimate Relationships	4 (3.2)	4 (3.2)	9 (7.3)	8 (6.5)	0.96**	1.00
Money	101 (81.5)	101 (81.5)	3 (2.4)	3 (2.4)	1.00**	1.00
Benefits	12 (9.7)	11 (8.9)	1 (0.8)	1 (0.8)	1.00**	1.00

**p < 0.01; * *p* < 0.05; K = Kappa; K_m_ = Maximum attainable kappa; K/K_m_ = Maximum agreement reached.

# Kappa coefficient could not be calculated because of insufficiently spread data.

### Comparing user and caregiver ratings of individual areas of needs

Similar to the inter-rater reliability found between professionals and users, the average K/K_m_ was 0.89, which indicates that 89% of the maximum possible agreement between users and caregivers was reached (see Table 5). All the areas of the CANE reached a K/K_m_ value over 0.60, indicating a good agreement between users and caregivers. Table 5 shows that users and caregivers reached 100% of the maximum possible agreement in the areas of looking after the home, deliberate self-harm, and intimate relationships. Similar to professionals, caregivers rated higher needs (met and unmet) than users in most of the sections of the CANE. They identified much a higher number of met needs for memory, looking after the home, money, drugs, physical health, and self-care; and a much higher number of unmet needs for daytime activities, company, psychological distress and eyesight and hearing.

**Table 5:** User and caregiver ratings of need in individual CANE areas

**User vs caregiver (n = 108)**	**User met need (%)**	**Caregiver met need (%)**	**User unmet need (%)**	**Caregiver unmet need (%)**	**K**	**K/ K**_**m**_
Accommodation	6 (5.6)	9 (8.3)	5 (4.6)	9 (8.3)	0.58**	0.79
Looking after home	73 (67.6)	93 (86.1)	1 (0.9)	1 (0.9)	0.51**	1.00
Food	81 (75.7)	91 (85.0)	0 (0.0)	3 (2.8)	#	#
Self-Care	36 (33.3)	62 (57.4)	0 (0.0)	6 (5.6)	#	#
Caring for another another	0 (0.0)	0 (0.0)	0 (0.0)	0 (0.0)	#	#
Daytime Activities Activities	20 (18.5)	35 (32.4)	14 (13.1)	46 (42.6)	0.24**	0.61
Memory	77 (71.3)	102 (94.4)	3 (2.8)	6 (5.6)	#	#
Eyesight/Hearing Hearing	31 (28.7)	35 (32.4)	11 (10.2)	22 (20.4)	0.66**	0.86
Mobility	28 (25.9)	41 (38.0)	2 (1.9)	6 (5.6)	0.64**	0.94
Continence	15 (13.9)	23 (21.3)	1 (0.9)	6 (5.6)	0.63**	0.97
Physical Health Health	51 (47.2)	72 (66.7)	5 (4.6)	5 (4.6)	0.59**	0.92
Drugs	51 (47.7)	79 (73.1)	2 (1.9)	5 (4.6)	0.44**	0.94
Psychotic Symptoms	6 (5.6)	10 (9.3)	3 (2.8)	6 (5.6)	0.61**	0.88
Psychological Distress	17 (15.7)	24 (22.2)	20 (18.5)	32 (29.6)	0.59**	0.84
Information	10 (9.3)	24 (22.2)	13 (12.0)	12 (11.1)	0.48**	0.67
Deliberate Self-harm Self-Harm	1 (0.9)	3 (2.8)	5 (4.6)	6 (5.6)	0.79**	1.00
Accidental Self-Harm Self-Harm	8 (7.4)	25 (23.1)	3 (2.8)	11 (10.2)	0.34**	0.87
Abuse/Neglect	2 (1.9)	10 (9.3)	2 (1.9)	2 (1.9)	#	#
Behaviour	3 (2.8)	7 (6.5)	0 (0.0)	5 (4.6)	#	#
Alcohol	2 (1.9)	3 (2.8)	0 (0.0)	3 (2.8)	#	#
Company	8 (7.4)	16 (14.8)	11 (10.2)	34 (31.5)	0.42**	0.91
Intimate Relationships	3 (2.8)	4 (3.7)	3 (2.8)	7 (6.5)	0.74**	1.00
Money	59 (54.6)	87 (80.6)	0 (0.0)	1 (0.9)	#	#
Benefits	10 (9.3)	5 (4.6)	0 (0.0)	1 (0.9)	#	#

**p < 0.01; * *p* < 0.05; K = Kappa; K_m_ = Maximum attainable kappa; K/K_m_ = Maximum agreement reached.

# Kappa coefficient could not be calculated because of insufficiently spread data.

## Discussion

Our study found that the most common unmet needs reported by caregivers were similar to those found in The Netherlands by van der Roest *et al*. [6], these being daytime activities (understood as any activity that allows persons with dementia to occupy themselves such as social, stimulation or leisure activities), company and psychological distress. However, van der Roest *et al*. [6] found that caregivers reported memory as one of the most frequent unmet need. This may relate to differences in service provision between the countries. For example, in the United Kingdom, people with dementia often have access to a community mental health team whereas in The Netherlands, services may be less systematised. Alternatively, it may be a result of UK participants being generally recruited from people with dementia known to services so that their memory needs had already been addressed. In both the United Kingdom and The Netherlands, there were services available to meet the needs that were unmet. There could be different reasons why services are not meeting the needs of people with dementia such as lack of suitability, lack of caregiver satisfaction with the services and/or refusal of services already offered. In line with the results of van der Roest *et al*. [6], the current study found that the most common unmet needs reported by people with dementia were psychological distress, daytime activities, company and information (about care and treatment). Whereas van der Roest *et al*. [6] found memory to be the second most common self-reported unmet need (11.9%), in this study memory was one of the less common unmet needs (3.2%). Memory was mostly (89.3%) met by receiving informal and formal help together. As mentioned previously, differences in identified needs may lie in differences of service provision and coordination.

The needs expressed by people with dementia in the review of von Kutzleben *et al*. [9] are in line with the most frequent unmet needs that we found. For example, the theme “emotions” refers mainly to negative feelings reported by people with dementia, such as anger, sadness, loneliness, confusion and worrying. All these feelings correspond to “psychological distress,” which, in our study, was the commonest unmet need reported by people with dementia. In the same way, the topics “losses and restrictions”, “maintaining normality”, and “dementia as a challenge for relationships”, which refer respectively to loss of meaningful activities and meaningful relationships, the need to continue doing the things they used to do and being useful for others, and also the loss of intimacy with the partner and potential conflicts with relatives, reflect what people with dementia reported in our study as unmet in the areas of “daytime activities” and “company”. It is a matter of concern that in our study, as well as in residential care [4], people with dementia reported most of the needs found by von Kutzleben *et al*. [9], van der Roest *et al*. [10] and de Boer *et al.*[11] as unmet (psychological distress, company, information, and daytime activities). If these areas are shown in the literature as those more reported and consequently more important from the point of view of the person with dementia, and they are yet unmet, it is essential that services and community organisations focus their resources on providing better care for improving those areas. This highlights the importance of involving people with dementia in their own care, of asking what kinds of support are more suitable for them, and of assessing the quality and appropriateness of the services provided.

### Comparing the needs among users, caregivers, and professionals

The most common needs were similar among people with dementia, caregivers, and the professionals, including memory, food, looking after the home, and money. Across all the areas of the CANE, the maximum possible agreement achieved was over 0.60, indicating good agreement between users, caregivers, and the professionals on identifying needs. The agreement was higher between professionals and caregivers than between people with dementia and caregivers and people with dementia and professionals. This could mean that the professionals took more account of the perspective of the caregiver than of the person with dementia, given that the levels of need are closer.

In agreement with previous studies [5,6,18], people with dementia reported different and significantly fewer needs (met and unmet) compared with their caregivers and the professionals. This could be explained by several reasons. In our study, no association was found between level of cognitive impairment (on the MMSE) and the number of unmet needs reported by people with dementia. However, awareness of cognitive impairment may be more important than its actual level since people who lack awareness of their problems may rate their quality of life as higher [19]. In a review of qualitative studies of people in the early stages of a dementia, Steeman *et al*. [20] found that some people with dementia ignored their difficulties as a way to deal with perceived threats such as loss of autonomy. One more plausible reason is that people with dementia had different priorities or concerns compared with their caregivers and professionals. For example, for people with dementia the most frequent unmet need was “psychological distress” whereas for caregivers and professionals this was the third most frequent, “daytime activities” being the commonest. It may also be that, having different priorities, people with dementia reported fewer needs than caregivers and professionals in the items of the CANE because not all the areas that people with dementia consider as important are covered by this instrument, such as areas of loss (“coping with disabilities”, “acceptance of dementia and help”, and “grief and frustrations about disabilities”), and self-esteem/self-image [10]. Depending on the aims of future research, the CANE may be used to complement other measures that include the topics mentioned.

“Information” (about the disease and support available) was the only unmet need rated higher by both caregivers and people with dementia (compared with professionals). This result is in accordance with other studies. In two reviews [9,21] it was found that people with dementia highly value the reception of continuous information during the progress of the disease, from the disclosure of the diagnosis to what services are available for them. Also, in a survey carried out among people with dementia living at home in the United Kingdom, Lakey *et al.*[22] found that 38% of people with dementia reported that they were not receiving any information or were getting some but not enough information about their condition. In addition, 68% of the persons with dementia received a diagnosis at least a year after the onset of the symptoms. These issues makes coping with the disease difficult for the person with dementia. A better provision of information might help people appraise their needs more accurately and help them to cope with the dementia in a more adaptive way, including a better use of services.

Discrepancies between people with dementia versus caregivers and professionals confirm the importance of including not only the professional and caregiver view about the needs of person with dementia but also their own views. It is important to notice that a lower agreement in perceived needs was found between people with dementia and their caregivers. This may have consequences in terms of seeking, acceptance, and compliance with treatment. It has been found that, when people with dementia perceive their subjective needs are not being taken into account by professionals, they report a bad communication and interaction with the health care system [9]. In contrast, when people with dementia and their caregivers feel that they are both taking part in their own care, the quality of life and self-esteem of people with dementia are improved [23,24]. Related to this, Lakey et al. found that 51% of people with dementia perceived they were not able, or were able only sometimes, to make decisions about their day-to-day lives. Also, 44% felt that professionals did not involve them, or that they did only sometimes, in decisions about their care and support. The CANE is a useful tool to assess different perspectives on needs. Using it would allow care practitioners to provide appropriate care packages that meet the needs of people with dementia by considering the scope for negotiation, flexibility and creativity.

In terms of limitations, the sample was drawn from those known to services and consequently the results cannot necessarily be generalised to people with dementia living at home who have not been in contact with services. Also, complete data from user, caregiver and professional assessments of needs were not available for every participant, limiting the power of the analysis, and this is likely to be due to the lack of a caregiver in some cases, but also the range in the severity of people with dementia leading to some being unable to complete the assessment of their own needs. The CANE is primarily a clinically-based assessment and a strength of this study was that all the researchers were experienced professionals in clinical psychology/old age psychiatry which is likely to improve the clinical validity of the results; previous work has shown that mental health nurses can also make accurate assessments. Bearing in mind that participants were known to services, there are a number of implications for service providers and commissioners. Many people lacked company and daytime activities and also had depressive symptoms suggesting that there should be better provision of social resources such as day centres and possibly more support (or respite provision) for caregivers (who rated these needs as more common). Also, unmet needs for eyesight/hearing were very common, suggesting that better assessment for and/or provision of glasses and hearing aids would have benefitted many people, especially as sensory problems have a major impact on communication in people with dementia.

## Conclusion

People with dementia reported fewer needs compared with the reports of their caregivers and the professionals. Thus, our hypothesis was confirmed. Although the common unmet needs reported by people with dementia, caregivers and professionals were almost the same, caregivers and professionals reported daytime activities as the most frequent unmet need whilst people with dementia considered psychological distress as the commonest unmet need. These results confirm the importance of assessing the needs of people with dementia by considering their own views. Further research is necessary to clarify the reason why different and fewer self-reported unmet needs were found, but the literature suggests that when people with dementia have been involved in their care and have felt a part of the decision-making process, there has been an improvement in their quality of life.

## Competing interests

The authors declare that they have no competing interests.

## Authors’ contributions

CMC designed the study, collected data, carried out the statistical analyses, wrote the paper, reviewed the manuscript and approved the final version. BW contributed to the interpretation of data, reviewed the manuscript and approved the final version. MO designed the study, contributed to the interpretation of data, reviewed the manuscript and approved the final version.

## Pre-publication history

The pre-publication history for this paper can be accessed here:

http://www.biomedcentral.com/1472-6963/13/43/prepub
